# Serum neutrophil gelatinase-associated lipocalin and recovery of kidney graft function after transplantation

**DOI:** 10.1186/1471-2369-15-123

**Published:** 2014-07-28

**Authors:** Maria E Hollmen, Lauri E Kyllönen, Jussi Merenmies, Kaija T Salmela

**Affiliations:** 1 Department of Medicine, Division of Nephrology, Helsinki University Hospital, Haartmaninkatu 4, PL 372, Helsinki 00029, HUS, Finland; 2 Department of Surgery, Division of Transplantation, Helsinki University Hospital, Helsinki, Finland; 3 Clinical Laboratory, Finnish Red Cross Blood Service, Helsinki, Finland

**Keywords:** Kidney transplantation, Delayed graft function, Serum NGAL, Point-of-care analysis

## Abstract

**Background:**

Neutrophil gelatinase-associated lipocalin (NGAL) is a marker for acute kidney injury. We studied whether serum NGAL predicts delayed graft function (DGF) and recovery of kidney function after transplantation.

**Methods:**

Serum NGAL was analyzed using commercial ELISA and point-of-care (POC) (Triage®, Biosite) methods. Serum samples were collected from 176 consecutive, deceased-donor kidney recipients just before transplant surgery and on day 1 and 14 after transplantation. The first 132 samples were analyzed with both methods and the remaining samples with the POC method.

**Results:**

The correlation between the ELISA and POC methods was 0.89, p < 0.0001 and hence the POC method was used for the remaining analyses. DGF was seen in 66/176 patients. Day 1 sNGAL was significantly higher in DGF (588 ng/ml, SD 189.6) compared to early graft function (355 ng/ml, SD 166.2, p < 0.0001) and this difference persisted on day 14. Day 1 sNGAL predicted DGF with an area under the curve (AUC) of 0.853 (CI 0.792-0.914, p < 0.0001). At the optimal cutoff level of 423 ng/ml the sensitivity was 87% and the specificity 77%. In a multivariate analysis, day 1 sNGAL emerged as an independent predictor of DGF. The sNGAL also predicted DGF lasting longer than 14 days with an AUC of 0.825 (CI 0.751-0.899, p < 0.0001). At the optimal cutoff level of 486 ng/ml, the sensitivity was 80% and specificity 75%.

**Conclusion:**

Serum NGAL predicts clinically significant DGF and is useful in the care of kidney transplant recipients.

## Background

Neutrophil gelatinase-associated lipocalin (NGAL) is a small, positively charged iron-carrier protein expressed at low levels in various epithelial cells (e.g. kidney, gastrointestinal tract, lungs) [1,2]. Due to its size and charge, NGAL is freely filtered through the glomerulus. Normal, steady state serum and urine NGAL concentration is approximately 20 ng/ml [3]. NGAL is markedly increased in the serum and urine of patients with acute kidney injury (AKI) [4-7]. Delayed graft function (DGF) is a form of AKI and is associated with complicated posttransplant recovery and an inferior 1-year outcome [8-15]. Prolonged DGF has been reported to severely impair the long-term prognosis of kidney transplantation [8,12]. The diagnosis of DGF is currently made on clinical grounds days after transplantation. However, we, and others, have shown that urine NGAL also predicts DGF after deceased-donor kidney transplantation [16-18]; moreover, there is data suggesting that measuring serum/plasma NGAL soon after transplantation is additionally valuable in predicting DGF [19-24]. NGAL has also been shown to predict kidney injury in liver transplant patients [25-27]. Despite these promising results, the use of NGAL has not yet been adopted in clinical transplantation.

The gold standard for NGAL analyses is immunoblotting. However, this method is time consuming and not readily available in clinical settings. Urine NGAL can also be measured using ELISA or a chemiluminescent microparticle immonoassay (ARCHITECT®, Abbott). When testing NGAL in the blood, ELISA or the recently introduced point-of-care (POC) fluorescence-based immunoassay (Triage®, Biosite) can be used. This POC test enables fast, bedside measuring of NGAL. However, data concerning the use of the POC method in NGAL analyses is still scarce.

Therefore, the aim of this study was to analyze how the POC method correlates with the ELISA method in measuring serum NGAL (sNGAL) and whether sNGAL predicts the occurrence and duration of DGF.

## Methods

### Study design and patient population

This study is parallel to our previous work on urine NGAL in the prediction of DGF [16]. We recruited 176 consecutive, adult, dialysis-dependent, deceased-donor kidney transplant recipients between August 2007 and August 2008. Here, we analyzed NGAL using the prospectively collected serum samples from this patient population. The Department of Surgery and the Ethics Committee at Helsinki University Hospital approved the study protocol. Written informed consent was obtained from the recipients before enrolment.

The primary outcome variable was the onset of graft function after transplantation, defined here, as it is most widely defined, as the need for dialysis during the first week after transplantation. However, in this classification, patients requiring one dialysis due to fluid overload or high potassium levels are also included in the DGF group. Conversely, there are DGF patients with fluent urine output from their native kidneys needing dialysis only after the first week and they are thus excluded from the DGF group. Therefore, to assess in more detail NGAL’s potential to predict DGF, we additionally performed a receiver operating characteristic (ROC) analysis using the DGF definition published by Halloran et al. consisting of plasma creatinine concentration >500 μmol/l throughout the first week, or oliguria of less than 1000 ml/24 hours for more than two days, or more than one dialysis session needed during the first week (28).

We collected the clinical data from the patients’ medical records and the Finnish Kidney Transplant Registry database, as previously described [16].

### Sample collection and NGAL analyses

A pretransplant (day 0) blood sample was taken upon arrival to the transplant unit. This sample was used to assess serum NGAL concentrations in patients with end-stage renal disease and as a reference value for the later time points. Posttransplant samples were collected in the morning following the transplant surgery (day 1) and 14 days after the transplantation (day 14). The samples were immediately processed and stored at -70°C.

We used a commercial ELISA kit (BioPortoDiagnostics, Gentofte, Denmark), as recommended by the manufacturer, for the NGAL analyses. The day 0, day 1, and day 14 samples were also analyzed using a point-of-care (POC) fluorescence immunoassay NGAL kit and device (Triage® Biosite, San Diego, California, USA), as recommended by the manufacturer. All measurements were performed in duplicate and blinded to the sample sources and clinical outcomes.

### Statistical analyses

SPSS software version 20.0 (SPSS Inc., Chicago, Illinois, USA) was used for the statistical analyses. All analyzed variables were tested for distribution. *T*-test and ANOVA were used for samples with normal distribution, and the Mann–Whitney U and Kruskal-Wallis tests for analyses of samples with skewed distribution. Chi-square and Fisher’s exact tests were employed in analyses of contingency tables. Bivariate correlations were analyzed using the Spearman correlation coefficient for non-parametric, and the Pearson correlation coefficient for parametric, measures of statistical dependence. To assess DGF predictors, a multivariate analysis was used. A ROC analysis was performed to assess NGAL’s potential to predict DGF and prolonged DGF. A p-value <0.05 was considered significant.

## Results

**Figure 1:**
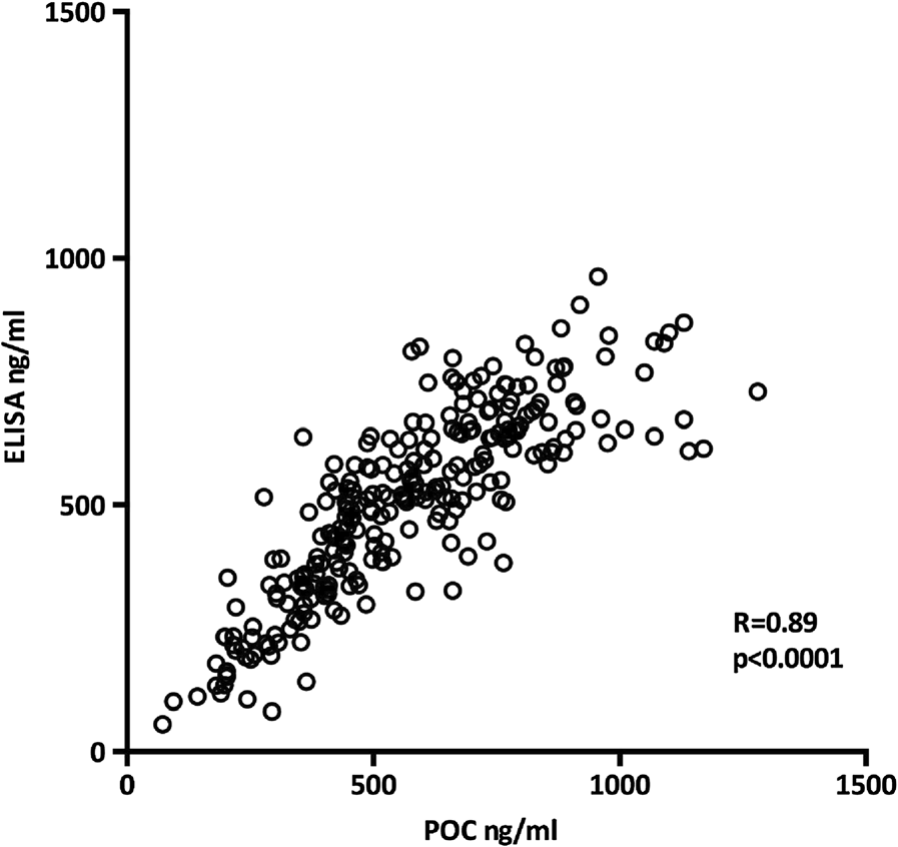
**The correlation of sNGAL values using the ELISA and POC methods.** The measurements were performed on the day 0 and day 1 samples of 132 kidney-transplant recipients.

Pretransplant serum samples from 132 kidney recipients and day 1 samples from 128 recipients were analyzed with both ELISA and POC. The corresponding ELISA and POC sNGAL values are shown in Figure 1. The mean sNGAL was 506 ng/ml (SD 188.7) measured with ELISA, and 536 ng/ml (SD 238.4) measured with POC. Their correlation was 0.89, p < 0.0001. Since the correlation was good we decided to use the sNGAL values measured using the more practical POC method for the clinical analyses in this study.

In recipients with no residual diuresis from their native kidneys, the mean pretransplant sNGAL was significantly higher (822 ng/ml, SD 193.5) than that of recipients with residual diuresis: the mean pre-transplant sNGAL was 697 ng/ml (SD 166.5) if the diuresis was 100–1000 ml/24 hours and 561 ng/ml (SD 164.4) if the diuresis was more than 1000 ml/24 hours (p < 0.0001). There was no significant difference between hemodialysis and peritoneal dialysis patients in this respect (data not shown). Pretransplant NGAL was not affected by recipient age, gender, underlying kidney disease, number of previous transplantations, mode or length of pretransplant dialysis, or pretransplant plasma creatinine (data not shown).

After transplantation, recipients with a urine output on day 1 of more than 1000 ml had significantly lower sNGAL levels (356 ng/ml, SD 168.1) compared to those with a urine output of less than 1000 ml (593 ng/ml, SD 185.7, p < 0.0001). Recipients with a decrease in plasma creatinine on day 1 compared to their pretransplant level had significantly lower sNGAL levels (382 ng/ml, SD 196.0) compared to those with no change or an increase in plasma creatinine on day 1 (547 ng/ml, SD 186.7, p < 0.0001). There were no significant differences in the other parameters studied (data not shown).

**Figure 2:**
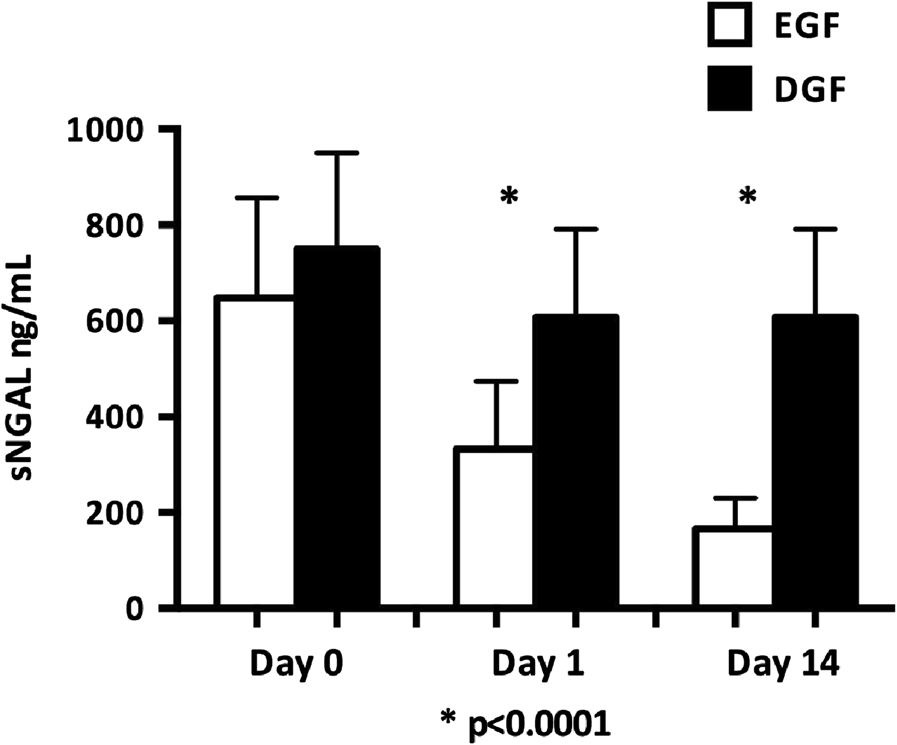
**The sNGAL concentration at day 0, day 1, and day 14 in early graft function (EGF) and delayed graft function (DGF) groups measured using the point-of-care (POC) method.** The results are expressed as means (+standard deviation, SD). DGF is defined as the need for dialysis during the first week after transplantation.

The patient and transplantation characteristics according to the onset of graft function are shown in Table 1. DGF was seen in 66/176 patients and their oliguria lasted a mean of 12.1 days (SD 7.0). When compared to the early graft function (EGF) group, the DGF group’s donors were older, expanded criteria donors [28] were more common, CIT longer, pretransplant hemodialysis more common than peritoneal dialysis, and time on pretransplant dialysis longer.Day 1 sNGAL was significantly higher in patients with DGF (588 ng/ml, SD 189.6) compared to those with EGF (355 ng/ml, SD 166.2, p < 0.0001) and this difference persisted on day 14 (Figure 2). Day 1 sNGAL correlated with the duration of DGF (R = 0.70, p < 0.0001). Day 1 sNGAL correlated with day 1 plasma creatinine (R = 0.64, p < 0.0001), day 3 plasma creatinine (R = 0.76, p < 0.0001), day 7 plasma creatinine (R = 0.69 p < 0.0001), day 1 urine output (R = 0.65, p < 0.0001), day 3 urine output (R = 0.51, p < 0.0001), and day 7 urine output (R = 0.39, p < 0.0001).

**Table 1:** Patient and transplantation characteristics

	**Early graft function (EGF) ** **n = 110 (62.5%)**	**Delayed graft function (DGF) ** **n = 66 (37.5%)**	**p-value**
**Mean recipient age years (SD)**	50.4 (12.8)	54.4 (13.2)	NS
**Gender, male (%)**	65 (59.1)	45 (68.2)	NS
**Re-transplantation (%)**	8 (7.3%)	7 (10.6%)	NS
**Mode of dialysis, hemodialysis (%)**	63 (57.3%)	51 (77.3%)	0.009
**Time on dialysis before transplantation, days (SD)**	788 (570.3)	953 (608.7)	0.029
**Mean donor age years (SD)**	49.1 (14.6)	56.2 (11.5)	0.001
**Expanded criteria donors**	33 (30.0%)	36 (54.5%)	0.001
**Mean cold ischemia time, hours (SD)**	21.3 (3.5)	22.9 (3.7)	0.003
**Day 1 mean plasma creatinine, μmol/L (SD)**	464 (218.4)	207 (114.4)	<0.0001
**Day 1 mean urine output, mL/24 hours (SD)**	2504 (1514.8)	502 (538.5)	<0.0001
**Number of rejections**	5 (4.5%)	5 (7.5%)	NS
**Mean eGFR, mL/min at 1 year (SD)**	66.2 (20.1)	54.8 (20.5)	<0.0001
**1-year patient survival**	99.1%	98.5%	NS
**1-year graft survival**	98.2%	89.4%	0.015

eGFR = estimated glomerular filtration rate calculated using the MDRD equation. SD = standard deviation. Delayed graft function defined according to the conventional definition: i.e. the need for dialysis during the first week after transplantation. Expanded criteria donor status defined according to Port et al. [28].

**Figure 3:**
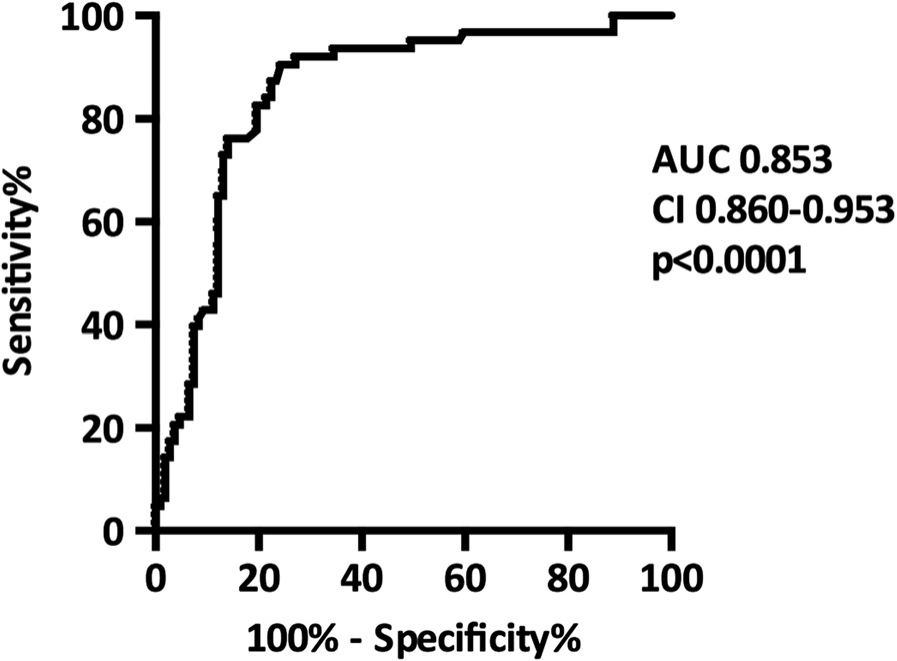
**The ROC-curve in predicting delayed graft function (DGF) defined as the need for dialysis during the first week after transplantation.** AUC, area under the curve; CI, confidence interval.

A ROC analysis was performed to assess the potential of day 1 sNGAL in predicting DGF. The area under the curve (AUC) was 0.853 (CI 0.792-0.914, p < 0.0001) (Figure 3). At the optimal cutoff level of 423 ng/ml, the sensitivity was 87% and the specificity 77% (the sensitivities and specificities for different cutoff levels are shown in Table 2). In comparison, urine output on day 1 predicted DGF with an AUC of 0.962 (CI 0.922-0.979, p < 0.0001). At the optimal cutoff level of 1035 ml/24 h, the sensitivity was 92% and the specificity 81%. Moreover, day 1 plasma creatinine predicted DGF with an AUC of 0.785 (CI 0.718-0.852, p < 0.0001). At the optimal cutoff level of 523 μmol/l, the sensitivity was 74% and the specificity 71%. The change in plasma creatinine from the pretransplant level to the day 1 level predicted DGF with an AUC of 0.769 (CI 0.696-0.843, p < 0.0001). At the optimal cutoff level of 68 μmol/l, the sensitivity was 76% and specificity 55%.

**Table 2:** The sensitivities and specificities at different cutoff levels for day 1 sNGAL in predicting delayed graft function (DGF)

**Day 1 sNGAL ng/ml**	**Sensitivity**	**Specificity**
71	1.00	0.00
199	0.97	0.11
302	0.95	0.41
423	0.87	0.77
607	0.40	0.93
852	0.11	0.98
1131	0.00	1.00

sNGAL = serum neutrophil gelatinase-associated lipocalin. Delayed graft function defined according to the conventional definition: i.e. the need for dialysis during the first week after transplantation.

**Figure 4:**
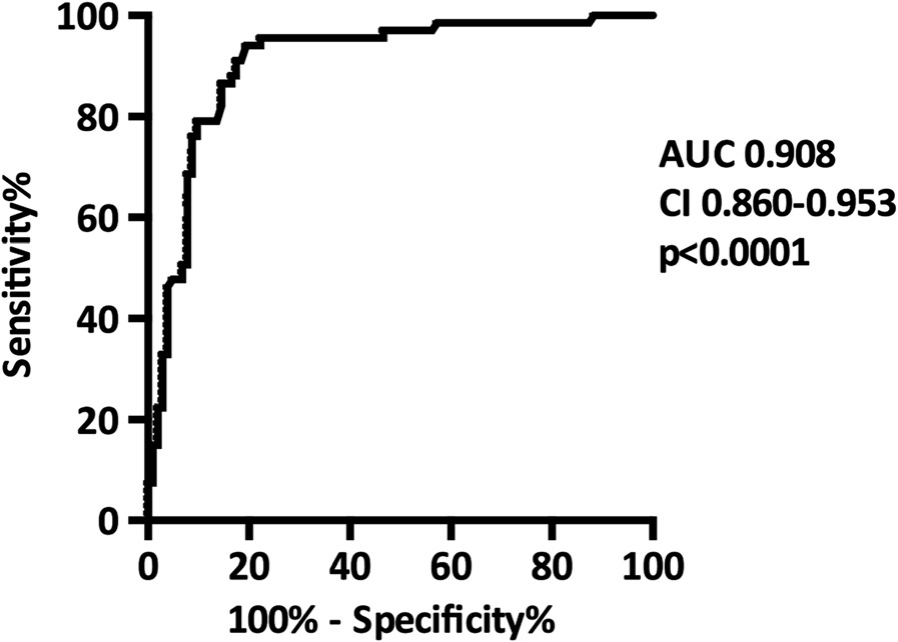
**The ROC-curve in predicting delayed graft function (DGF) defined using the Halloran et al. criteria for DGF**[29]**.** AUC, area under the curve; CI, confidence interval.

Additionally, we repeated the ROC analysis for sNGAL on day 1 after classifying the patients according to the criteria for DGF by Halloran et al. [29]; the AUC for day 1 sNGAL in predicting DGF was 0.908 (CI 0.860-0.955, p < 0.0001) (Figure 4). At the optimal cutoff level of 426 ng/ml, the sensitivity was 91% and the specificity 83%. The sensitivities and specificities for different cutoff levels are shown in Table 3.

**Table 3:** The sensitivities and specificities at different cut off levels for Day 1 sNGAL in predicting delayed graft function (DGF) using the Halloran definition for DGF

**Day 1 sNGAL ng/ml**	**Sensitivity**	**Specificity**
71	1.00	0.00
201	0.99	0.14
323	0.97	0.51
426	0.91	0.83
568	0.51	0.92
794	0.15	0.98
1131	0.00	1.00

sNGAL = serum neutrophil gelatinase-associated lipocalin. Delayed graft function defined according to the Halloran criteria [29].

Altogether, 10 patients were classified differently into the DGF and EGF groups depending on whether the conventional or the Halloran criteria for DGF were used (Table 4). All of the patients who otherwise had prompt function but required one dialysis (classified as DGF by conventional criteria and EGF by the Halloran criteria) had day 1 sNGAL less than 400 ng/ml. On the other hand, those with poorly declining plasma creatinine but with no dialysis performed (classified as EGF by conventional criteria and DGF by the Halloran criteria) had day 1 sNGAL higher than 400 ng/ml.

**Table 4:** Subgroup of 10 kidney transplantations categorized differently depending on the used DGF definition

**Patient**	**DGF definition**	**Conventional**	**UOP 1d**	**Δ creatinine d0-d1**	**sNGAL 1d**
	**Halloran**				
1	EGF	DGF	1750	-98	374
2	EGF	DGF	1710	-264	198
3	EGF	DGF	1670	-60	221
4	DGF	EGF	430	-272	604
5	DGF	EGF	2090	-74	722
6	DGF	EGF	2060	-319	759
7	DGF	EGF	1330	-67	971
8	DGF	EGF	100	-83	657
9	DGF	EGF	2350	-11	451
10	DGF	EGF	1030	+88	706

EGF = early graft function, DGF = delayed graft function, UOP = urine output, Δcreatinine d0-d1 = change in plasma creatinine from pretransplant level to day 1, sNGAL = serum neutrophil gelatinase-associated lipocalin.

In a multivariate analysis, we assessed the effect on the occurrence of DGF of: donor age, donor creatinine, donor eGFR, expanded criteria donor status, CIT, recipient age, mode and length of dialysis before transplantation, change in plasma creatinine from day 0 to day 1, day 1 urine output, and day 1 sNGAL. Day 1 sNGAL and day 1 urine output emerged as independent predictors of DGF, irrespective of the DGF definition used (Tables 5 and 6).

**Table 5:** Multivariate analysis of risk factors for delayed graft function (DGF) defined using the conventional definition

	**p-value**
**Donor age (years)**	0.115
**Donor plasma creatinine (μmol/L)**	0.232
**Donor eGFR (mL/min)**	0.348
**Expanded criteria donors**	0.434
**Cold ischemia time (hours)**	0.364
**Recipient age (years)**	0.504
**Mode of dialysis (hemodialysis or peritoneal dialysis)**	0.058
**Time on dialysis before transplantation (days)**	0.488
**Change in plasma creatinine from pretransplant to day 1**	0.792
**Recipient day 1 urine output (mL)**	<0.0001
**Recipient day 1 sNGAL (ng/mL)**	0.019

eGFR = estimated glomerular filtration rate calculated using the MDRD equation, sNGAL = serum neutrophil gelatinase-associated lipocalin. Delayed graft function defined according to the conventional definition: i.e. the need for dialysis during the first week after transplantation. Expanded criteria donor status defined according to Port et al. [28].

**Table 6:** Multivariate analysis of risk factors for delayed graft function (DGF) defined using the definition of Halloran et al.

	**p-value**
**Donor age (years)**	0.707
**Donor plasma creatinine (μmol/L)**	0.622
**Donor eGFR (mL/min)**	0.751
**Expanded criteria donors**	0.929
**Cold ischemia time (hours)**	0.063
**Recipient age (years)**	0.994
**Mode of dialysis (hemodialysis or peritoneal dialysis)**	0.102
**Time on dialysis before transplantation (days)**	0.721
**Change in plasma creatinine from pretransplant to day 1**	0.586
**Recipient day 1 urine output (mL)**	<0.0001
**Recipient day 1 sNGAL (ng/mL)**	<0.0001

eGFR = estimated glomerular filtration rate calculated using the MDRD equation, sNGAL = serum neutrophil gelatinase-associated lipocalin. Delayed graft function defined according to Halloran et al. [28]. Expanded criteria donor status defined according to Port et al. [28].

The day 1 serum samples were drawn the morning after transplant surgery, a mean 11.8 hours (SD 5.2) after the reperfusion of the graft. The time interval between reperfusion and this day 1 sample varied from 2 hours to 24 hours; it was 6 hours or less in 18 cases, 7 to 12 hours in 82 cases, and more than 12 hours in 70 cases. DGF was predicted by sNGAL well in all time interval groups, but the predictive power was best in the group were the sample was drawn less than 6 hours after reperfusion (Table 7).

**Table 7:** Time from reperfusion to day 1 serum sampling

**Sample time after reperfusion**	**Mean sNGAL ng/ml EGF (SD)**	**Mean sNGAL ng/ml DGF (SD)**	**p-valueDGF vs EGF**	**ROC-analysis AUC, p-value, CIoptimal cut-off value, sensitivity, specificity**
<6 hours	n = 9	n = 9	0.002	1.00, p = 0.001, CI 1.00-1.00
	278 (78.8)	687 (239.4)		436 ng/ml, 100%, 100%
7 to 12 hours	n = 46	n = 36	<0.0001	0.864, p < 0.0001, CI 0.783-0.946
	365 (159.5)	594 (169.2)		420 ng/ml, 94%, 73%
>12 hours	n = 47	n = 23	<0.0001	0.920, p < 0.0001, CI 0.838-0.999
	321 (125.0)	526 (187.9)		420 ng/ml, 91%, 83%

EGF = early graft function, DGF = delayed graft function, AUC = area under the curve, CI = confidence interval. Delayed graft function defined according to the conventional definition: i.e. the need for dialysis during the first week after transplantation.

To assess whether sNGAL predicted a prolonged period of posttransplant oliguria, we divided the patients according to the onset of graft function: <14 days (n = 150) or ≥14 days (Prolonged DGF) (n = 26). There were significantly more expanded criteria donors, day 1 creatinine was higher, and day 1 urine output lower in the prolonged DGF group compared to the group with onset of function <14 days (Table 8). The mean day 1 sNGAL was significantly higher in the prolonged DGF group (655 ng/ml, SD 217.6), compared to those with the onset of graft function before day 14 (404 ng/ml, SD 183.2, p < 0.0001). Day 1 sNGAL predicted prolonged DGF with an AUC of 0.825 (CI 0.751-0.899, p < 0.0001). At the optimal cutoff of 486 ng/ml, the sensitivity was 80% and the specificity 75%. In multivariate analysis, day 1 urine output and day 1 sNGAL emerged as independent predictors for prolonged DGF (Table 9).

**Table 8:** Patient and transplantation characteristics according to the prolonged onset of graft function

	**Before 14 days**	**At/later 14 days**	**p-value**
**N**	150	26	
**Mean age, years (SD)**	51.8 (12.7)	52.6 (15.5)	NS
**First transplantation**	138/150 (92.0%)	24/26 (92.3%)	NS
**Mode of dialysis, hemodialysis**	95 (22.9%)	19 (73.1%)	NS
**Mean time on dialysis, days (SD)**	842 (614.1)	899 (410.2)	NS
**Donor age, years (SD)**	51.0 (14.3)	56.3 (SD 10.9)	NS
**CIT, hours (SD)**	21.7 (3.7)	23.2 (3.4)	NS
**ECD donor status**	53 (35.3%)	16 (61.5%)	0.008
**Day 1 creatinine, μmol/L (SD)**	508 (219.4)	676 (224.6)	0.002
**Day 1 urine output, mL (SD)**	1983 (1594.0)	392 (446.6)	<0.0001
**1-year graft survival**	148 (98.7%)	19/26 (73.1%)	<0.0001
**1-year patient survival**	149 (99.3%)	25 (96.2%)	NS

SD = standard deviation, CIT = cold ischemia time, ECD = expanded criteria donor, defined according to Port et al. [28].

**Table 9:** Multivariate analysis of risk factors for prolonged delayed graft function (DGF)

	**p-value**
**Donor age (years)**	0.405
**Donor plasma creatinine (μmol/L)**	0.867
**Donor eGFR (mL/min)**	0.113
**Expanded criteria donors**	0.119
**Cold ischemia time (hours)**	0.416
**Recipient age (years)**	0.653
**Mode of dialysis (hemodialysis or peritoneal dialysis)**	0.861
**Time on dialysis before transplantation (days)**	0.867
**Change in plasma creatinine from pretransplant to day 1**	0.930
**Recipient day 1 urine output (mL)**	0.001
**Recipient day 1 sNGAL (ng/mL)**	0.042

eGFR = estimated glomerular filtration rate calculated using the MDRD equation, sNGAL = serum neutrophil gelatinase-associated lipocalin. Expanded criteria donor status defined according to Port et al. [28].

## Discussion

We have recently shown that NGAL measured in the urine of deceased organ donors is an independent risk factor for prolonged DGF, suggesting that donor urine NGAL levels reflect the quality of the donor kidney and its ability to tolerate ischemia-reperfusion injury during transplantation [30]. Additionally, we, and others, have found the urine NGAL of kidney transplant recipients to be an early marker of DGF.

Serum/plasma NGAL has also been reported to predict DGF in various studies. In one of these studies, NGAL levels were analyzed using the POC method [21], whereas the other studies had used the ELISA or the ARCHITECT method. The first study by Kusaka et al. found that NGAL levels were significantly higher in DGF patients than those with immediate graft function, and in cases requiring hemodialysis after transplantation the serum NGAL levels were >400 ng/ml [19]. The study by Lebkowska et al. found that NGAL correlated with serum creatinine, cystatin C, and urine output. Serum NGAL levels were and remained significantly higher after transplantation in the DGF group (mean 419 ng/ml) compared to the EGF group (mean 308 ng/ml) [20]. Bataille et al. found that pretransplant plasma NGAL was high in all patients (mean 453 ng/ml). After transplantation, plasma NGAL was significantly higher in the DGF group (mean 571 ng/ml) compared to the transplant patients with immediate graft function (mean 242 ng/ml). Indeed, plasma NGAL predicted DGF at 12 hours after transplantation with an AUC of 0.97. NGAL concentration >400 ng/ml predicted DGF with a sensitivity of 94% and specificity of 89% [21].

Furthermore, Lee et al. found that serum NGAL was higher in transplant patients with DGF and that day 1 serum NGAL predicted DGF with an AUC of 0.86, sensitivity of 79% and specificity of 50% [22]. In their more recent study, Kusaka et al. found that sNGAL measured using the ELISA method correlated well with sNGAL measured using the ARCHITECT method. The sNGAL concentration decreased markedly and rapidly after transplantation in patients with immediate or slow graft function, but not in those with DGF or prolonged DGF. They found that day 1 sNGAL 500 ng/ml predicted the need for dialysis with an AUC of 0.99, sensitivity of 91% and specificity of 97%. Day 1 sNGAL of 700 ng/ml predicted prolonged DGF with an AUC of 0.93, sensitivity of 82%, and specificity of 83%. In their study, day 1 sNGAL correlated significantly with urine output, duration of DGF, and serum creatinine on day 5 [23]. Mahdavi-Mazdeh et al. found that serum NGAL levels were significantly higher in the DGF and slow graft function groups compared to the early graft function group. NGAL was able to identify patients with slow graft function from early graft function unlike creatinine. They found that NGAL measured at 24 hours after transplantation best predicted DGF with an AUC of 0.82 [24]. The findings in these studies, although examining relatively small (n = 16 to 67) and heterogeneous kidney transplant patient populations (deceased-donor, living donor, donation after cardiac death), are in line with our results: serum NGAL predicts DGF and an NGAL level of >400 ng/ml gives the best sensitivity and specificity.

The ELISA test is well established and routinely used for research. However, it is time consuming and prone to human error. When we began this project, only the ELISA test was available; this test was thus used for the NGAL analyses of the first 132 study patients. During the course of the study a commercial POC kit for analyzing NGAL levels from blood became available and was subsequently used for the NGAL analyses of all the 176 study patients. As the correlation between the NGAL measurements using these two methods was acceptable and the POC method is better suited for clinical work, we used the POC test results in the evaluation of serum NGAL as a predictor of DGF. Pretransplant sNGAL was measured to investigate the serum NGAL levels in dialysis-dependent patients, and these values were used as a reference for sNGAL after transplantation. The only variable significantly affecting pretransplant sNGAL was residual diuresis. Anuric or severely oliguric patients had substantially higher sNGAL levels than patients with ample diuresis. It is probable that, as the tubules no longer function, their uptake of NGAL is limited and thus it is excreted in the urine, causing the sNGAL concentration to decrease.

Furthermore, day 1 sNGAL predicted DGF well in the ROC analysis. It also correlated with the length of DGF and predicted prolonged DGF well. In the multivariate analysis, day 1 sNGAL and day 1 urine output emerged as independent predictors of DGF. In fact, day 1 urine output predicted DGF better than sNGAL. Usually, diuresis is the easiest, and cheapest, method to assess the onset of graft function. However, there are patients who have copious diuresis and still experience DGF; in contrast, there are recipients with only moderate diuresis but rapidly decreasing plasma creatinine without the need for posttransplant dialyses.

Analysis of day 1 sNGAL enables a diagnosis of DGF and prolonged DGF days before it is possible clinically. Although there is no specific treatment for DGF, recognizing the patients who have an increased risk of developing DGF helps clinicians to optimize postoperative care and avoid nephrotoxic treatments and interventions.

Our study does have certain limitations. First, it was a single center study. Further, we had not standardized the timing of the day 1 sampling, and the number of blood samples taken very soon after reperfusion was low. The confounding effects of surgery, dialysis, anesthesia, and different medications on sNGAL are also not known. Serum, instead of the recommended plasma/whole blood, was used for the POC analyses and we only studied one biomarker instead of a panel of biomarkers. However, our study also had several strengths. It is a nationwide study of 176 consecutive, adult, deceased-donor kidney recipients and, so far, this is the largest study reporting sNGAL levels in kidney transplant recipients. We analyzed the sNGAL levels at the most significant time point after transplantation (day 1) using two different methods and we analyzed the data using two different, widely acknowledged definitions of DGF.

## Conclusions

Our study clearly demonstrated that day 1 sNGAL predicted DGF well, even in cases where such a diagnosis would be impossible on the basis of clinical signs alone. Furthermore, day 1 sNGAL predicted prolonged DGF, which affects the long-term success of transplantation. The best performance of sNGAL in predicting DGF was seen in the small group of cases with a timeframe of six hours or less from reperfusion to blood sampling. In the future, this finding should be confirmed with standardized blood sampling. Therefore, on the basis of the results of this study, sNGAL clearly has a place in the postoperative surveillance of kidney transplant patients.

## Abbreviations

AKI: Acute kidney injury; AUC: Area under the curve; CIT: Cold ischemia time; DGF: Delayed graft function; EGF: Early graft function; eGFR: Estimated glomerular filtration rate; NGAL: Neutrophil gelatinase-associated lipocalin; sNGAL: Serum neutrophil gelatinase associated lipocalin; POC: Point-of-care; SD: Standard deviation.

## Competing interests

The authors declare that they have no competing interests.

## Authors’ contributions

MH performed the NGAL analyses, collected the data, analyzed the data, and wrote the manuscript. LK participated in designing the study and analyzing data. JM participated in designing the study, and revised the manuscript. KS participated in designing the study, acquired funding, and participated in writing the manuscript. All authors read and approved the final manuscript.

## Pre-publication history

The pre-publication history for this paper can be accessed here:

http://www.biomedcentral.com/1471-2369/15/123/prepub

## References

[B1] Axelsson M Bergenfeldt M Ohlsson K Studies of the release and turnover of a human neutrophil lipocalin Scand J Clin Lab Invest 1995 55 577 588 8633182 10.3109/00365519509110257

[B2] Cowland JB Borregaard N Molecular characterization and pattern of tissue expression of the gene for neutrophil gelatinase-associated lipocalin for humans Genomics 1997 45 17 23 9339356 10.1006/geno.1997.4896

[B3] Paragas N Qiu A Hollmen M Nickolas TL Devarajan P Barasch J NGAL-Siderocalin in kidney disease Biochim Biophys Acta 1823 2012 1451 1458 10.1016/j.bbamcr.2012.06.014 PMC3664277 22728330

[B4] Mishra J Ma Q Prada A Mitsnefes MM Zahedi K Yang J Barasch J Devarajan P Identification of neutrophil gelatinase-associated lipocalin as a novel early urinary biomarker for ischemic renal injury J Am Soc Nephrol 2003 14 2534 2543 14514731 10.1097/01.asn.0000088027.54400.c6

[B5] Mishra J Dent C Tarabishi R Mitsnefes MM Ma Q Kelly C Ruff SM Zahwedi K Shao M Bean J Mori K Barasch J Devarajan P Neutrophil gelatinase-associated lipocalin (NGAL) as a biomarker for acute renal injury following cardiac surgery Lancet 2005 365 1231 1238 15811456 10.1016/S0140-6736(05)74811-X

[B6] Mori K Lee HT Rapaport D Drexler IR Foster K Yang J Schmidt-Ott KM Chen X Li JY Weiss S Mishra J Cheema FH Markowitz G Suganami T Sawai K Mukoyama M Kunis C D’Agati V Devarajan P Barasch J Endocytic delivery of lipocalin-siderophore-iron complex rescues the kidney from ischemia-reperfusion injury J Clin Invest 2005 115 610 621 15711640 10.1172/JCI23056 PMC548316

[B7] Schmidt-Ott KM Mori K Li JY Kalandadze A Cohen DJ Devarajan P Barasch J Dual action of neutrophil gelatinase-associated lipocalin J Am Soc Nephrol 2007 18 407 413 17229907 10.1681/ASN.2006080882

[B8] Giral-Classe M Hourmant M Cantarovich D Dantal J Blancho G Daguin P Ancelet D Soulilou JP Delayed graft function of more than six days strongly decreases long-term survival of transplanted kidneys Kidney Int 1998 54 972 978 9734625 10.1046/j.1523-1755.1998.00071.x

[B9] Kyllönen LE Salmela KT Eklund BH Halme LE Höckerstedt KA Isoniemi HM Mäkisalo HJ Ahonen J Long-term results of 1047 cadaveric kidney transplantations with special emphasis on the initial of graft function and rejection Transpl Int 2000 13 122 128 10836648 10.1007/s001470050295

[B10] Sola R Alarcon A Jimenez C Osuna A The influence of delayed graft function Nephrol Dial Transplant 2004 19 iii32 iii37 15192133 10.1093/ndt/gfh1012

[B11] Humar A Johnson EM Payne WD Wrenshall L Sutherland DE Najarian JS Killingham KJ Matas AJ Effect of initial slow graft function on renal allograft rejection and survival Clin Transplant 1997 11 623 627 9408697

[B12] Dominguez J Lira F Rebolledo R Troncoso P Aravena C Ortiz M Gonzalez R Duration of delayed graft function is an important predictor of 1-year serum creatinine Transplant Proc 2009 41 131 132 19249496 10.1016/j.transproceed.2008.10.028

[B13] Yarlagadda SD Coca SG Formica RN Jr Poggio ED Parikh CR Association between delayed graft function and allograft and patient survival: a systemic review and meta-analysis Nephrol Dial Transplant 2009 24 1039 1047 19103734 10.1093/ndt/gfn667

[B14] Johnston O O’Kelly P Spencer S Donohoe J Walshe JJ Little DM Hickey D Conlon PJ Reduced graft function (with or without dialysis) vs immediate graft function – a comparison of long-term renal allograft survival Nephrol Dial Transplant 2006 21 2270 2274 16720598 10.1093/ndt/gfl103

[B15] Humar A Ramcharan T Kandaswamy R Gillingham J Payne WD Matas AJ Risk factors for slow graft function after kidney transplants; a multivariate analysis Clin Transplant 2002 16 425 429 12437622 10.1034/j.1399-0012.2002.02055.x

[B16] Hollmen ME Kyllönen LE Inkinen KA Lalla ML Salmela KT Urine Neutrophil gelatinase-associated lipocalin is a marker of graft recovery after kidney transplantation Kidney Int 2011 79 89 98 20861824 10.1038/ki.2010.351

[B17] Hall IE Yarlagadda SG Coca SG Wang Z Doshi M Devarajan P Han WK Marcus RJ Parikh CR IL-18 and Urinary NGAL predict dialysis and raft recovery after kidney transplantation J Am Soc Nephrol 2010 21 189 197 19762491 10.1681/ASN.2009030264 PMC2799276

[B18] Parikh CR Jani A Mishra J Ma Q Kelly C Barasch J Edelstein CL Devarajan P Urine NGAL and IL-18 are predictive biomarkers for delayed graft function following kidney transplantation Am J Transplant 2006 6 1639 1645 16827865 10.1111/j.1600-6143.2006.01352.x

[B19] Kusaka M Kuroyanagi Y Mori T Nagaoka K Sasaki H Maruyama T Hayakawa K Shiroki R Kurahashi H Hoshinaga K Serum neutrophil gelatinase-associated lipocalin as a predictor of organ recovery from delayed graft function after kidney transplantation from donors after cardiac death Cell Transplant 2008 17 129 134 18472448 10.3727/000000008783907116

[B20] Lebkowska U Malyszko J Lebkowska A Koc-Zorawska E Lebkowski W Malyszko JS Kowalewski R Gacko M Neutrophil geltainase-associated lipocalin and cystatin c could predict renal outcome n patients undergoing kidney allograft transplantation Transplant Proc 2009 41 154 157 19249501 10.1016/j.transproceed.2008.10.092

[B21] Bataille A Abbas S Semoun O Bourgeois E Marie O Bonnet F Resche-Rigon M Abboud I Losser MR Jacob L Plasma neutrophil gelatinase-associated lipocalin in kidney transplantation and early renal function prediction Transplantation 2011 92 1024 1030 21956199 10.1097/TP.0b013e318230c079

[B22] Lee EY Kim MS Park Y Kim HS Serum neutrophil gelatinase-associated lipocalin and interleukin-18 as predictive biomarkers for delayed graft function after kidney transplantation J Clin Lab Anal 2012 26 295 301 22811364 10.1002/jcla.21520 PMC6807575

[B23] Kusaka M Iwamatsu F Kuroyanagi Y Nakaya M Ichino M Marubashi S Nagano H Shiroki R Kurahashi H Hoshinaga K Serum neutrophil associated lipocalin during the early postoperative period predicts the recovery of graft function after kidney transplantation from donors after cardiac death J Urol 2012 187 2261 2267 22503046 10.1016/j.juro.2012.01.033

[B24] Mahdavi-Mazdeh M Amerian M Abdollahi A Hatmi ZM Khatami MR Comparison of serum neutrophil gelatinase-associated lipocalin (NGAL) with serum creatinine in prediction of kidney recovery after renal transplantation Int J Org Transplant Med 2012 3 176 182 PMC4089298 25013643

[B25] Portal AJ McPhail MJ Bruce M Coltart I Slack A Sherwood R Heaton ND Shawcross D Wendon JA Heneghan MA Neutrophil gelatinase-associated lipocalin predicts acute kidney injury in patients undergoing liver transplantation Liver Transpl 2010 16 1257 1266 21031541 10.1002/lt.22158

[B26] Feldkamp T Bienholz A Kribben A Urinary neutrophil gelatinase-associated lipocalin (NGAL) for the detection of acute kidney injury after orthopic liver transplantation Nephrol Dial Transplant 2011 5 1456 1458 21486868 10.1093/ndt/gfr146

[B27] Wagener G Minhaz M Mattis FA Kim M Emond JC Lee HT Urinary neutrophil gelatinase-associated lipocalin as a marker of acute kidney injury after orthopic liver transplantation Nephrol Dial Transplant 2011 26 1717 1723 21257679 10.1093/ndt/gfq770 PMC3145384

[B28] Port FK Bragg-Gresham JL Metzger RA Dykstra DM Gillespie BW Young EW Delmonico FL Wynn JJ Merion RM Wolfe RA Held PJ Donor characteristics associated with reduced graft survival: an approach to expanding the pool of kidney donors Transplantation 2002 74 1281 1286 12451266 10.1097/00007890-200211150-00014

[B29] Halloran PF Aprile MA Farewell V Ludwin D Smith EK Tsai SY Bear RA Cole EH Fenton SS Cattran DC Early function as the principal correlate of graft survival. A multivariate analysis of 200 cadaveric renal transplants treated with a protocol incorporating antilymphocyte globulin and cyclosporine Transplantation 1988 46 223 228 3043779

[B30] Hollmen ME Kyllönen LE Inkinen KA Lalla ML Merenmies J Salmela KT Deceased donor neutrophil gelatinase-associated lipocalin and delayed graft function after kidney transplantation Crit Care 2011 15 R121 21545740 10.1186/cc10220 PMC3218974

